# Adding noise can reduce response biases in addition to improving perceptual performance

**DOI:** 10.1016/j.isci.2025.113227

**Published:** 2025-07-29

**Authors:** Elise Rowe, Jeroen J.A. van Boxtel

**Affiliations:** 1 Discipline of Psychology, Faculty of Health, University of Canberra, Bruce, ACT, Australia; 2 Turner Institute for Brain and Mental Health, School of Psychological Sciences, Monash University, Melbourne, VIC, Australia

**Keywords:** Psychology

## Abstract

“Stochastic resonance” (SR) is a phenomenon whereby adding an “optimal” amount of noise can *improve* perceptual capabilities. Although this performance enhancement is important, in many tasks we also want to reduce biases, such as the tendency to respond “absent” to an infrequent stimulus. To address this, we designed a perceptual task where we presented six possible letters (“C,”“B,”“H,” “O,”“E,” and “U”) at threshold levels. We induced a response bias by manipulating the frequency of the consonants and vowels at 80% (frequent stimuli) versus 20% (rare stimuli) and induced SR using Gaussian visual luminance noise. We observed SR-based improvements in performance (accuracy and d’) across 22 observers at low to intermediate noise levels. Additionally, response biases (i.e., criterion) decreased due to an increase in correctly identified rare stimuli. Our results suggest that low-to-moderate noise can improve performance and reduce response biases, with potential implications for real-world tasks (such as cancer screening) where target (i.e., tumor) occurrence is rare.

## Highlights

• The addition of noise can increase perceptual accuracy and reduce response bias
• Improvements occurred at more noise levels for rare stimuli compared to frequent stimuli
• Low-level noise could possibly be used to reduce response biases in real-world tasks

## Graphical abstract

**Figure:**
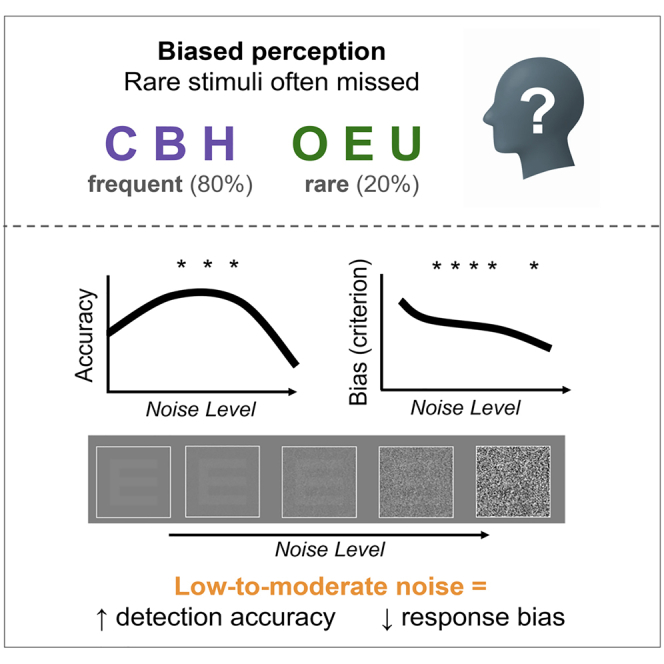

## Introduction

Noise, or random signal variability, is present in all biological and computational systems and can arise from both internal and external sources (see^1^). External noise refers to the signal-unrelated information in the environment, while internal noise is generated via the computations required to process irrelevant sensory information at cellular and neuronal levels.^2^ Generally, noise is considered harmful, leading to a lower signal-to-noise ratio and decreases in perceptual capabilities.^3^^,^^4^ Strikingly, adding an “optimal” amount of external noise can actually improve perceptual performance in specific circumstances, a phenomenon known as stochastic resonance (SR;^5^^,^^6^). SR occurs when a subthreshold sensory signal is boosted above the perceptual threshold through the injection of a precise amount of external noise. This type of SR-based performance enhancing has been shown in the perception of weak auditory,^7^ visual^8^ and tactile^9^ stimuli (for review see^10^), and can improve vision in individuals with severe visual impairments.^11^

While SR has been shown for perceptual performance, human behavior is often also influenced by biases, which in turn can lead to suboptimal performance. These biases can present in individuals as response (or decision) bias to, for example, respond “no” to the presence of an infrequent (or rare) signal.^12^^,^^13^ Such biases may arise, for example, in radiography, where the presence of cancerous tumors is rare. Indeed, 30%–70% of detected breast cancers can be retrospectively detected in earlier mammograms.^14^^,^^15^ Thus, if SR can contribute to the decrease of such biases, then this may allow us to more precisely identify the target signal within the noise and improve detection performance overall.

To measure how an observer deals with the noisiness (or uncertainty) of sensory signals, the Signal Detection Theory (SDT;^16^) framework is often used. SDT describes how an observer determines what they are currently perceiving given some level of ambiguity (e.g., stimulus or noise). For example, when a radar operator is determining whether a stimulus on the screen is an enemy plane or a flock of birds, they must determine the strength of the evidence for either option.^17^ It follows that the greater the strength of the evidence, the easier it is for the observer to determine whether it is present. In addition, an individual also has their own “decision criterion” (or bias) that describes how much evidence is required before they respond “present.” For example, if an enemy plane is frequently flying overhead, then that individual will be more likely to think an ambiguous stimulus is a plane rather than a flock of birds. The “optimal” level of bias (or criterion) maximizes the outcomes of a hit (that is, correctly identifying an enemy plane) and correct rejection (that is, correctly identifying a flock of birds), with the best strategy leading to the greatest reward (or the lowest loss/cost). In other words, the decision criterion refers to how often an individual can accept misidentifying a target.

As the criterion is a direct reflection of the decisions that individuals make (when determining if a signal is present or not, or deciphering between two alternatives), it is flexible in that it can change depending on the situation or the task requirements; a process known as criterion shifting.^12^^,^^18^ Thus, for all decisions, the bias can be both perceptual or decisional and will change depending on the individual or the circumstance. Interestingly, given an equal number of two possible alternative choices (for example, signal present or absent), individuals have been shown to vary widely from conservative (i.e., a tendency to respond “absent”) to liberal (or tendency to respond “present”),^19^ and thus show suboptimal performance due to bias.

However, in everyday life, it is uncommon for our choice alternatives to be present in equal proportions. Past research has shown that when presented with vastly uneven proportions (often called base rates and signifying a difference in the frequency of the two alternatives), individuals tend to be biased toward the more frequent occurrence.^12^^,^^13^ Ideally, all rare events are correctly identified; however, high ambiguity and sub-threshold signal strength can hamper this process. While methods such as training, feedback, or monetary compensation can be employed to reduce such biases,^20^^,^^21^^,^^22^ these methods take long periods of time or may not be practically implemented in real-life situations (see^23^^,^^24^). Thus, SR might offer the potential to increase observer accuracy and reduce response bias quickly and simply via the introduction of small amounts of noise during detection tasks. Accordingly, the current study aimed to invoke SR in the presence of decision biases (induced by uneven base rates) to determine whether the decision biases in observer behavior can be overcome using the SR phenomenon. Such a finding could lead to applications that induce SR-like responses to increase accuracy regardless of the uneven frequencies (or payoffs) that are encountered in everyday life (e.g., in cancer screening).

## Results

**Figure 1:**
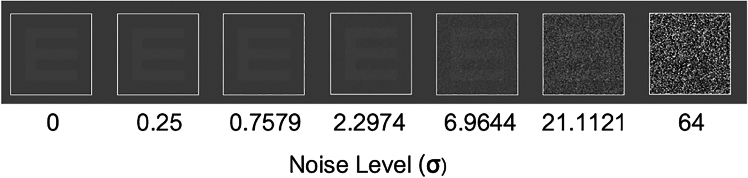
Gaussian pixel noise was presented with standard deviations (σ) ranging from 0 to 64 in 7 equally spaced steps (in log space) The letters could be consonants (B, C, or H) or vowels (E, O, U) and were shown in the Sloan font. Note that the letter luminance is increased for visualization purposes.

We investigated whether invoking SR in the presence of decision biases could lead to a reduction (or removal) of response bias. To measure an observer’s bias, we used Signal Detection Theory (SDT;^16^), which allows us to analyze perceptual decisions in terms of the independent components of sensitivity (d’), and criterion (i.e., bias). Perceptual decisions involved identifying letters, presented in visual noise. Letters consisted of both consonants (“C,” “B,”and “H”) and vowels (“O,” “E,” and “U”). Decision biases were induced by setting uneven base rates of these letter categories at 80/20 consonants versus vowels (or vowels versus consonants). Gaussian pixel external noise was presented with standard deviations (σ) ranging from 0 to 64 in 7 equally spaced steps (in log space): 0, 0.25, 0.76, 2.30, 6.96, 21.11, and 64σ (Figure 1 and see Table 1). Participants had to identify the letter they observed. We defined accuracy as responding with the correct letter category (i.e., a consonant or a vowel) on a given trial. We estimated d’ and criterion by considering a hit as responding with a rare letter (when the letter shown was a rare letter) and a miss as responding with a frequent letter. Similarly, correct rejections were defined as responding with a frequent letter (when the letter shown was a frequent letter) and false alarms as responding with a rare letter. In doing so, we kept with the traditional application of SDT that uses the criterion to examine response biases (see STAR Methods and^16^ for further explanation).

**Table 1:** Experimental block design

Order	Block	Parameters	Trials	Stimulus
0	Practice	Zero noise, luminance level = “easy” at 132	18	6 letters x 3 repetitions
1	Block 1 - QUEST	Zero noise, variable luminance for 70% accuracy	50	Randomly selected letter on each trial
2	Block 2	Zero noise, no instructions about letter base rates	48	80% consonants, 20% vowels
	Criterion checked to determine bias toward consonants or vowels			
3	Block 3	Zero noise, informed 80/20 base rates	48	80/20 or 20/80 consonants or vowels, depending on the participant’s bias
4	Block 4	8 noise levels, informed 80/20 base rates	240	80/20 or 20/80 consonants or vowels, depending on bias
5	Block 5	Repeat block 4	240	Repeat block 4

### Stochastic resonance effect on accuracy

We first established that the addition of noise led to conventional measures of SR, that is, the highest accuracy and d’ are reached at non-zero levels of visual noise. For accuracy, we further split these results into the rare or frequent letter categories (based on what that participant had been shown) and combined these at the group level. For d’, this split could not be made, as d’ was calculated by comparing the rare and frequent letters in a 2AFC manner.

**Figure 2:**
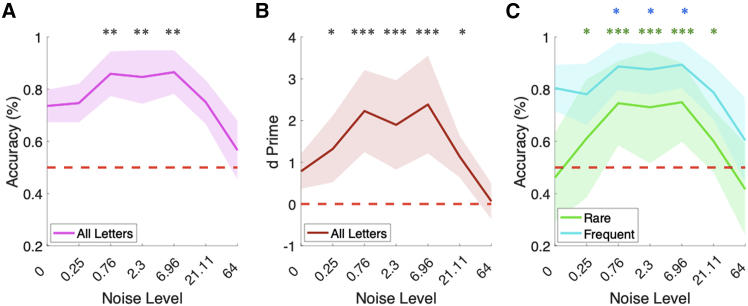
Stochastic resonance improves letter recognition accuracy and sensitivity (A and B) We observed a significant increase in task accuracy and d’ comparing zero-noise (0σ) baseline with 0.76σ, 2.3σ, and 6.96σ. In addition, we observed a significant increase in d’ at 0.25σ and 21.11σ. (C) When splitting the letters into rare or frequent, we observed a significant increase in accuracy for the frequent letters at 0.76σ, 2.3σ, and 6.96σ and for the rare letters at 0.25σ, 0.76σ, 2.3σ, 6.96σ, and 21.11σ. Error bars show standard deviation. N.B: ∗ = *p* < 0.01, ∗∗ = *p* < 0.001, ∗∗∗ = *p* < 0.0001.

As shown in Figure 2, for all measures, we observed the “inverted U-shape curve” which is the signature of SR. We found a significant SR-effect of the addition of noise on task accuracy (Figure 2; one-way repeated measures ANOVA over noise level, *F(6,147)* = 31.66, *p* = 3.02 × 10^−24^). Post hoc tests comparing accuracy at each noise level against the baseline condition of zero-noise (using one-tailed t-tests and FDR correction for multiple comparisons) showed a significant increase in accuracy from zero noise (M = 73.6%, ±6%) to noise levels 0.76σ (*M* = 85.9%, ±9%, *p*_*corr*_ = 9.00 × 10^−7^, d = 1.49), 2.30σ (*M* = 84.7%, ±10%, *p*_*corr*_ = 2.47 × 10^−6^, d = 1.34) and 6.96σ (*M* = 86.5%, ±8%, *p*_*corr*_ = 4.95 × 10^−7^, d = 1.56). We observed a similar effect on d’ in that we observed a significant main effect of noise level (one-way repeated measures ANOVA over noise levels, *F(*6,147) = 9.94, *p* = 3.38 × 10^−9^). Post hoc t-tests showed a significant increase in d’ from zero noise (M = 1.0 ± 0.6) at the noise levels of 0.25σ (M = 1.6 ± 1.0, pcorr = 0.0053, d = 0.61), 0.76σ (*M* = 2.1, ±0.8, *p*_*corr*_ = 9.69 × 10^−7^ d = 1.66), 2.30σ (*M* = 2.1, ±1.0, *p*_*corr*_ = 5.80 × 10^−5^, d = 1.28), 6.96σ (*M* = 2.1, ±0.8, *p*_*corr*_ = 9.60 × 10^−7^, d = 1.84) and 21.11σ (M = 1.5 ± 0.4, pcorr = 0.0058, d = 0.41).

When looking at accuracy for frequent and rare letters separately, we observed a main effect of noise level on accuracy for frequent letters (one-way repeated measures ANOVA, *F(6,147)* = 18.49, *p* = 5.99 × 10^−16^). Post hoc t-tests showed that, compared to baseline (M = 79.0%, ±10%), there was significant increase in accuracy at noise level 0.76σ (M = 88.3%, ±9%, *p*_*corr*_ = 0.0004, d = 0.84), 2.3σ (M = 87.6% ± 10%, pcorr = 0.0003, d = 0.72) and 6.96σ (*M* = 89.2%, ±9%, *p*_*corr*_ = 0.0003, d = 0.91). In addition, for the rare letters, we again observed a main effect of noise level on accuracy (one-way repeated measures ANOVA, *F(6,147)* = 13.00, *p* = 9.18 × 10^−12^). Post hoc t-tests showed that, compared to the zero-noise baseline (M = 46.6% ±17%), there was significant increase in accuracy at the same noise levels as for all letters: 0.76σ (*M* = 73.0%, ±16%, *p*_*corr*_ = 7.27 × 10^−7,^ d = 1.60), 2.30σ (*M* = 72.3%, ±21%, *p*_*corr*_ = 1.56 × 10^−5^, d = 1.52) and 6.96σ (*M* = 74.3%, ±14%, *p*_*corr*_ = 2.46 × 10^−7^, d = 1.62). In addition, we observed a significant increase in accuracy at 0.25σ (*M* = 58.8%, ±23%, *p*_*corr*_ = 0.0002, d = 0.84) and 21.11σ (*M* = 60.1%, ±12%, *p*_*corr*_ = 0.0003, d = 0.78). Finally, when we directly compared the accuracy at each noise level for the rare versus frequent stimuli, we found there was a significantly higher accuracy for the rare letters at zero noise (*p* = 3.41 × 10–6, d = 2.53), 0.25σ (*p* = 0.02, d = 0.94), 0.76σ (*p* = 0.001, d = 1.07), 2.30σ (*p* = 0.003, d = 0.87), 6.96σ (*p* = 0.0009, d = 1.15), 21.11σ (*p* = 0.0001, d = 1.66) and 64σ (*p* = 0.01, d = 1.10). These results show that SR occurred for accuracy and d’ (of all letters combined) at noise levels 0.76σ, 2.3σ, and 6.96σ, and additionally at the very low noise level of 0.25σ and higher noise level of 21.11σ for the rare letters and d’ (likely driven by this increase in accuracy for the rare letters).

### Stochastic resonance effect on response biases

**Figure 3:**
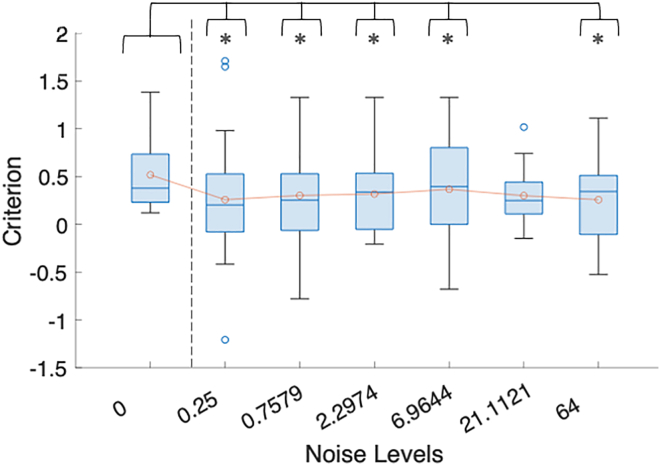
Stochastic resonance leads to a reduction in response bias (criterion) We compared the criterion (whiskers showing 1.5× the interquartile range) at each noise level against the zero-noise baseline criterion (itself determined to be significantly above zero) and found this was significantly reduced at 0.25σ, 0.76σ, 2.30σ, 6.96σ, and 64σ. N.B: ∗ = *p* < 0.05.

To determine how the response bias (or criterion) shifted in the presence of noise, we first ensured that our method had resulted in participants favoring the frequent letter category, as evidenced by a positive criterion value.^16^ Indeed, in the zero noise condition, the mean criterion was 0.52 ± 0.4, indicating a significant bias toward responding with one of the frequent stimuli (compared to 0, *t*(21) = 6.46, *p* = 2.11 × 10^−6^, Cohen’s *d* = 1.95; see Figure 3). Bayesian statistics, comparing the criterion against 0, found the natural logarithm of the Bayes Factor (lnBF_10_) to equal 9.11. Note that an lnBF_10_ of 5 represents very strong evidence for H_1_ (i.e., that the criterion was different from zero), 3 to 5 strong evidence for H_1_, between 3 and 1 positive evidence for H_1,_ and 0 to 1 weak (or anecdotal) evidence for H_1_.^25^^,^^26^

We used the individual zero-noise criterion values as the “baseline” criterion against which any changes induced by the addition of noise were compared (using simple t-tests, False Discovery Rate, FDR, corrected for multiple comparisons, Figure 3). We found a significant reduction in the criterion compared to baseline at almost all noise levels at which SR was observed: 0.25σ (M = 0.26 ± 0.7, p_corr_ = 0.0154, d = 0.48, lnBF_10_ = 1.912), 0.76σ (M = 0.30 ± 0.5, p_corr_ = 0.038, d = 0.49, lnBF_10_ = 0.8040), 2.3σ (M = 0.32 ± 0.4, p_corr_ = 0.0154, d = 0.51, lnBF_10_ = 1.778), 21.11σ (M = 0.30 ± 0.3, p_corr_ = 0.0146, d = 0.34, lnBF_10_ = 2.75) and 64σ (M = 0.26 ± 0.4, p_corr_ = 0.0105, d = 0.65, lnBF_10_ = 2.59 despite observing no SR effects or increases in d’ at this noise level). Thus, we could conclude that response biases were reduced in the presence of SR (and when accuracy fell to chance-level at 64σ). Importantly, we highlight that our results show independence between performance (i.e., accuracy) and the criterion. Firstly, when comparing zero-noise to 0.25σ at the group level, we find that accuracy does not change, but the criterion is significantly reduced. Secondly, to support these conclusions, we ran a post-hoc Bayesian correlation analysis between these two factors and found lnBF_10_ = 0.22, meaning there exists only weak evidence for a correlation. This independence is important as it suggests that reductions in the bias are genuine and not driven by changes in d’.

## Discussion

We aimed to invoke SR in the presence of decision biases to determine whether such biases in observer behavior can be overcome using the SR phenomenon. For this, we designed a perceptual task where we presented six possible letters at threshold visibility levels (set at 70% accuracy using the QUEST staircase procedure). We induced a response bias by manipulating the frequency of the consonants and vowels (or vowels and consonants) at 80% versus 20% and induced SR using Gaussian visual luminance noise. We found the first evidence that SR can reduce response biases at low to moderate noise levels (specifically between 0.25σ and 21.11σ). We also replicated previously observed SR effects on accuracy when examining all letter stimuli (specifically, at low to intermediate noise levels between 0.76σ and 6.96σ; see^11^) and additionally showed SR at the lowest external noise level of 0.25σ up to the moderate noise level of 21.11σ when we examined the rare letters alone. Interestingly, the response bias was significantly reduced over a broad range of noise levels. While improvements in accuracy could influence bias by altering hit and false alarm rates, we show that shifts in decision bias occur independently of changes in sensitivity (d') and that the SR effect influences decision-making itself rather than perceptual sensitivity, in line with the theoretical independence of d’ and criterion in SDT. Our results suggest that the SR effects observed for the rare letters are a genuine case of the reduction in response bias for the rarely occurring stimuli at this low noise level. Therefore, noise can reduce response biases due to uneven base rates of occurrence.

Reducing biases through the addition of small amounts of external noise could prove invaluable in situations such as baggage checks or cancer screening where individuals are biased toward responding “absent” to a rare stimulus (see^27^). Indeed, current statistics suggest that around 10–20% of baggage searches,^28^ 20% of lung cancers,^29^ and 70% of breast cancers^30^ are missed during routine scanning despite the experience of trained professionals. Recent studies suggest that the low frequency of occurrence is a major contributor to the high miss rates during cancer screening^27^^,^^30^ along with unconscious biases such as prior reports or previous diagnoses.^31^ The findings of the current study suggest that, in addition to potentially increasing accuracy at detecting infrequent (or rare) stimuli, the accompanying biases might also be reduced.

When interpreting the results from this study, there are two noteworthy considerations. The first is our choice to redefine our 6AFC into a 2AFC task by grouping responses according to their category. This was motivated by the knowledge that: (1) binarised responses are typical for SDT and (2) individuals use category-level information to group stimuli^32^^,^^33^ – and we explicitly instructed our participants to do so. A reduction in category-level bias is relevant to real-world situations (such as in cancer screening or radiology) where tumor appearances can differ but their category-level features (i.e., cancerous tissue) are paramount. In addition, our stimulus set was explicitly designed so that the two most confusable stimuli were those between the letter categories (i.e., E vs. B), minimizing the chance that this information was used to “guess” the correct category. Future studies could further test these mechanisms by explicitly requiring individuals to make a 2AFC response based on the stimulus category instead of identity. The second consideration is that the observed reduction in response bias at the highest noise level (64σ) was accompanied by a drop in accuracy to chance-level. Such effects do not reflect SR processes influencing decision bias. Instead, at this highest noise level, the noise likely overwhelms perceptual decision making and results in more neutral responses, even despite individuals being aware of the 80/20% letter category split. Future work could directly test this by manipulating decision strategies or by incorporating confidence ratings to determine if participants are merely guessing at the highest noise levels.

Future studies should also look at inducing other types of cognitive biases such as those encountered using monetary and dietary rewards^34^ or unconscious biases such as own-race bias^35^ or gender bias.^36^ Furthermore, the examination of whether SR could reduce decision biases such as confirmation bias, the anchoring effect, framing bias or loss aversion bias would be useful in a multitude of everyday situations. Indeed, if SR could be used in these important real-world situations to reduce bias, then such findings could dramatically improve social situations such as those encountered during police work, in teaching situations, or even during customer relationship management. Our work is the first step in that direction and shows the promise of the approach in a perceptual task.

In considering these options for future research, it is important to highlight some limitations of the current works. Namely, that the generalizability of such findings will need to be tested in these other real-world situations in which SR might be added to improve performance and reduce bias. Secondly, the recruited sample in the current study was all aged at an average of 22 years, while experienced baggage handlers and radiographers are often aged much older.^28^^,^^30^ Thus, another important question for future research is whether the same findings can be replicated in older and more expert samples, as these factors influence SR mechanisms.^37^ Furthermore, factors such as fatigue and stress, especially in tasks such as baggage handling or cancer screening where sustained attention is required, should be investigated and accounted for. Finally, when we induced our decision bias, we used uneven base rates of the stimulus categories when a number of other factors (as described above), such as personal history, learning rates, or monetary incentives, can also influence biases.^38^ It would be informative for future research to determine whether SR can overcome all types of biases.

### Conclusion

We designed a perceptual task to determine whether optimal levels of noise can reduce response biases (in addition to task accuracy) via a response known as SR. For this, we presented six possible letters at threshold levels and induced a response bias by manipulating the frequency of the consonants and vowels (or vowels and consonants) at 80% versus 20%. Furthermore, we induced SR using Gaussian visual luminance noise at one of six possible levels and compared performance against the zero-noise condition. As expected, based on past experiments, we observed SR-based improvements in performance (i.e., accuracy and d’) when comparing low-to-intermediate noise levels against the no noise conditions. SR occurred for task accuracy at more noise levels (both higher and lower) for the rare stimuli than for the frequent stimuli. We found that response biases (i.e., criterion) decreased at low-to-moderate (but not zero) noise levels, due to an increase in correctly identified rare stimuli. Our results suggest that noise can reduce response biases, which could have important implications on real tasks (such as cancer screening) where target (i.e., tumor) occurrence is relatively rare.

### Limitations of the study

To summarize the limitations of the current study: (1) The generalizability of these findings should be tested in real-world settings such as baggage handling or cancer screening, where SR may enhance performance and reduce bias. (2) As our sample was quite young (with an average age of 22 years old), future research should examine whether these effects hold in older and more expert populations. (3) Furthermore, the effects of fatigue and stress (which are critical in sustained attention tasks) also warrant investigation. (4) Lastly, since our induced bias was based on uneven stimulus base rates, future studies should explore whether SR can alleviate biases driven by other bias-inducing factors such as personal history, learning rates, or monetary incentives.

## Resource availability

### Lead contact

Further information and requests for resources should be directed to and will be fulfilled by the Lead Contact: Elise Rowe (elise.rowe@monash.edu).

### Materials availability

This study did not generate unique reagents.

### Data and code availability

• Data: Data are available in the following OSF repository: https://osf.io/skw7u/.
• Code: Analysis code can be found in the following OSF repository: https://osf.io/skw7u. Code to run the experimental paradigm can be found in the following GitHub repository: https://github.com/egrowe/SR_letter_task.
• Other: All other relevant materials are available upon request.

## Acknowledgments

The authors wish to thank the participants from the University of Canberra for their involvement in the study. We also gratefully acknowledge the reviewers for their valuable feedback that improved the article. This research was funded by the 10.13039/100015539Australian Government through an Australian Research Council Discovery Project (project number DP220100406) awarded to JJAVB.

## Author contributions

Conceptualization, J.V.B.; methodology E.R. and J.V.B..; investigation, E.R.; Writing – Original Draft, E.R.; writing – review and editing, E.R. and J.V.B.; funding acquisition, J.V.B..; resources, J.V.B..; supervision, E.R. and J.V.B.

## Declaration of interests

The authors declare that we have no competing interests.

## STAR★Methods

### Key resources table

**Table:** 

REAGENT or RESOURCE	SOURCE	IDENTIFIER
**Software and algorithms**		

Analysis code and deposited data	Open Science Framework Repository	https://osf.io/skw7u
Experimental code	GitHub Repository	https://github.com/egrowe/SR_letter_task
MATLAB 2021a	MathWorks	MathWorks, Inc. (2021). MATLAB (Version 9.10.0.1613233) [Software]. Natick, Massachusetts
Psychtoolbox3	Brainard^39^ and Pelli^40^	Brainard, D. H. (1997). The Psychophysics Toolbox. Spatial Vision, 10(4), 437–442. Pelli, D. G. (1997). The VideoToolbox software for visual psychophysics: Transforming numbers into movies. Spatial Vision, 10(4), 437-442

### Experimental model and study participant details

#### Subjects

Twenty-two subjects (age range: 18-37 years; M = 22.0years ± +/- 3 years, 14 females and 8 males) participated in the experiment (after the exclusion of 6 individuals, see below for further details). We selected this sample size based on an *a priori* power estimation that determined at least N = 19 were required to observe a medium effect (0.6) at alpha = 0.05 with 80% power. All participants had self-reported normal (or correct-to-normal) vision, no history of neurological or psychiatric disorder, and no previous head trauma resulting in unconsciousness. We note that, while information on the participants' sex was recorded, this study was not designed or powered to assess the effects of gender or sex on our study outcomes. We therefore did not conduct subgroup analysis and note this as a limitation of the current study. All participants were provided with information about the task requirements prior to participating and were informed that they could withdraw from the study at any time. Each participant provided written consent in accordance with The University of Canberra Human Ethics Committee guidelines.

### Method details

#### Experimental setup

The experimental paradigm was programmed using the Psychtoolbox3 extension^39^^,^^40^ for MATLAB2021a^41^ and displayed on a gamma-corrected Dell LCD monitor (60 Hz, 1920 × 1080 pixels) at a viewing distance of 57 cm without the aid of a chinrest.

#### Visual stimuli

We modified a paradigm previously shown to evoke SR effects in healthy individuals^42^^,^^43^ and the vision impaired.^11^ In our version of this paradigm, we presented one of six possible letters (C, B, H, O, E, U) at the centre of the screen. These letters were selected as we required an equal number of consonants and vowels to enable manipulation of the response bias (criterion) via unequal base rates (see below). In addition, we wanted each letter to be easily confusable with another from the opposite category (e.g., C for O, E for B and H for U). This was done to increase the task difficulty and ensure that correct responses were due to participants perceiving the letters and not making an informed guess.

We presented the letter stimuli for 1 second on a grey background (i.e., 127 for each R, G, B value) within a 100 × 100 pixel square (at 57 cm viewing distance). Each letter was shown at font size 67 (∼85 pixels or ∼2.3 degree visual angle) in the Sloan typeset that is commonly used in visual acuity tests. The luminance of the letters (gamma corrected) was determined in our first experimental block using the adaptive QUEST staircase procedure^44^ set to 70% accuracy (see below). If participants were not within +/- 10%, of this accuracy level in Block 2 (see below), the QUEST procedure was repeated, as per.^45^ No external noise was used in the QUEST block.

#### Noise stimuli

In the experimental blocks, where we tested the effect of external noise on accuracy and response biases (i.e., blocks 4 and 5), we introduced dynamic zero-mean Gaussian luminance noise at each pixel within the 100 × 100 letter presentation square. The noise had standard deviations (σ) ranging from 0 to 64 and we used 7 equally spaced steps (in log space): 0, 0.25, 0.76, 2.30, 6.96, 21.11 and 64σ. The noise was refreshed at 60Hz.

#### Experimental paradigm

The experiment comprised 5 blocks and a practice block (for summary see Table 1) and the total run time was around 45 minutes. At the beginning of each block, participants were informed of the task requirements verbally and were reminded via on-screen instructions prior to the first trial. In the practice block, participants were presented with 18 trials at an ‘easily discriminable’ luminance (no feedback was provided) that resulted in near 100% accuracy for every participant. Block 1 utilised the QUEST staircase procedure (see above) in which one letter was randomly selected on each trial (50 trials in total) and the task difficulty (in the form of the luminance level) was titrated to achieve 70% (±10%) accuracy using a 6-alternative-forced-choices method (i.e., “which letter did you see?”). The luminance level determined by the end of this block was used for all letter stimuli in the remaining blocks.

In Block 2 there were 48 zero-noise trials that were used to obtain the participant’s accuracy and response bias (or criterion, see below). In this block, 80% of the trials were a consonant with an equal proportion of each letter. We reasoned here that participants would be biased towards responding with the more common consonant as has been shown in the past.^12^^,^^13^ Participants were not informed of the 80/20 consonant/vowel base rate. After Block 2 concluded, we examined the participant’s criterion to determine whether they were more biased toward responding with a consonant or a vowel.

In Block 3, we manipulated each participant’s bias from Block 2 by switching the 80/20 proportion of letters toward their preferred letter category to determine whether we could enhance this bias. For example, an individual with a bias towards responding with a vowel, despite only being present 20% of the time in Block 2 would then be presented with 80% vowel trials in the subsequent blocks. For Block 3, the same conditions were repeated as Block 2, except that we informed participants that the trials would be presented with an 80/20 (or 20/80) consonant to vowel ratio prior to commencing. Thus, after Block 3, we obtained a measure of accuracy and the response criterion given the knowledge of uneven base rates. The criterion from this block was used to exclude participants (N = 6 for a total of N = 22) who did not show a bias (of any size) toward the frequent stimuli. We did this to ensure all participants were responding to our manipulation of letter frequency with the expected bias and allowed us to interpret the effects of the addition of noise on accuracy, d’ and criterion in subsequent blocks.

In Block 4 and 5, we added the dynamic Gaussian (external) noise at six possible non-zero levels. To ensure our stimulus presentation conditions were relative to a no noise baseline condition within the same block, we additionally included a zero-noise condition. Block 4 contained 240 trials (30 trials at each noise level, randomized order) with an 80/20 split of consonant/vowels or vowels/consonants (depending on the participant’s bias determined in Block 2). Participants were informed of the uneven base rates in this block. Finally, Block 5 was a repeat of Block 4 to increase the number of trials per noise level to 60.

### Quantification and statistical analyses

#### Data analysis

We were interested in determining whether introducing noise could: (1) induce SR effects for at-threshold perceptual judgments of the letter stimuli and (2) independently reduce response biases. We measured participant accuracy and d’ (or sensitivity to distinguish signal from noise independent of bias^16^; in selecting the appropriate letter category (vowel, consonant) on a trial-by-trial basis. That is, we converted our analysis of the 6-AFC behavioural task into a 2-AFC task by considering responses correct if the letter choice was within the same category (not only the same letter). We did not alter the physical properties of the stimuli or their visibility. In doing this, we kept with the traditional application of SDT that uses the criterion to examine response biases in a 2AFC manner (see below).

For this analysis, we first estimated the number of hits (H), misses (M), false alarms (FA) and correct rejections (CR; as per.^12^ We estimated the accuracy using the formula:

(H+CR)/(H+M+CR+FA)

We then calculated the hit rate (HR) and false alarm rate (FAR) using the following:

HR=H/(H+M)

FAR=FA/(CR+FA)

We estimated d’ using the following where z equals the z-score:

$$d^{\prime }=z\left(HR\right)-z\left(FAR\right)$$

Finally, we estimated criterion using the following:

c=−0.5∗z(HR)∗z(FAR)

Finally, to examine our primary research questions, we examined the mean accuracy, d’ and criterion values obtained at ‘baseline’ (i.e., the zero-noise level condition) in Blocks 4 and 5 (combining across all trials), and compared them to those obtained at each noise level to examine our primary research questions. This was also done to be more in line with real-life situations where individual targets are different, but a category of items needs to be found (i.e. a tumour, not a particular shape).

We used ANOVAs to determine if there was a main effect of noise level when looking at accuracy and d’ because these were continuous dependent variables where we wanted to test for overall differences between the noise levels. For post-hoc examination of these results, we used pairwise t-tests (one-tailed) adjusted using FDR correction as these tests have high sensitivity at detecting significant findings whilst also keeping Type 1 errors in check. For the criterion, we first examined the zero noise ‘baseline’ condition (in Blocks 4 and 5) per participant and used a simple t-test against 0 to establish whether a response bias was present. We included Bayesian statistics at this step to show the strength of the evidence that this (if any) bias was present. Next, we applied the same post-hoc methods as those used to examine accuracy and d’. That is, we examined the criterion shift by comparing against the zero-noise ‘baseline’ using pairwise t-tests and FDR correction. See results for all details relating to these analyses.

#### Measuring SR effects

To measure the SR effects at the group-level, we determined the mean accuracy and d’ values for the group at each noise level. We then used a one-way repeated measures ANOVA to determine if there was a main effect of noise level. Post-hoc multiple comparison FDR-corrected t-tests were used to compare the accuracy and d’ at each noise level against the zero-noise ‘baseline’. By definition, SR would be present if we observed a significant increase in task accuracy at a non-zero level of noise compared to the baseline (i.e., compared to the zero-noise condition in Blocks 4 and 5).

#### Measuring bias

To determine how the response bias (or criterion) shifted in the presence of SR, we first ensured that our method had resulted in participants favouring the frequent letter category, as evidenced by a positive criterion value.^16^ For this, we calculated the average criterion in the zero-noise condition across all trials in Blocks 4 and 5 (combined) and compared this value, per participant, against the unbiased criterion value of zero (using a one-way t-test). We used these zero-noise criterion values as the ‘baseline’ criterion against which any changes at each of the noise levels were compared. Next, we examined whether this criterion was significantly reduced at each noise level using the same methods as were applied to accuracy and d’.
